# Use of intraoperative parathyroid hormone measurements during parathyroidectomy to predict postoperative parathyroid hormone levels in patients with renal hyperparathyroidism: meta-analysis

**DOI:** 10.1093/bjsopen/zrab151

**Published:** 2022-02-15

**Authors:** Dirk-Jan van Beek, Stina Fredriksson, Stefanie Haegele, Marco Raffaelli, Philipp Riss, Martin Almquist

**Affiliations:** Department of Endocrine and Sarcoma Surgery, Skåne University Hospital, Lund, Sweden; Department of Clinical Sciences Lund, Lund University, Lund, Sweden; Department of Endocrine Surgical Oncology, University Medical Centre Utrecht, Utrecht, The Netherlands; Department of Endocrine and Sarcoma Surgery, Skåne University Hospital, Lund, Sweden; Department of Nephrology, Skåne University Hospital, Malmö, Sweden; Department of Surgery, Medical University of Vienna, Vienna, Austria; Division of Endocrine and Metabolic Surgery, Fondazione Policlinico Universitario Agostino Gemelli IRCCS, Università Cattolica del Sacro Cuore, Rome, Italy; Department of Surgery, Medical University of Vienna, Vienna, Austria; Department of Endocrine and Sarcoma Surgery, Skåne University Hospital, Lund, Sweden; Department of Clinical Sciences Lund, Lund University, Lund, Sweden

## Abstract

**Background:**

Several studies have reported on the use of intraoperative parathyroid hormone (ioPTH) measurements during parathyroidectomy (PTX) for renal hyperparathyroidism (rHPT), but there is no consensus on whether it is helpful and, if so, what protocol should be used. Therefore, the literature was systematically reviewed to assess a correlation between ioPTH and early postoperative parathyroid hormone (PTH) levels in patients undergoing PTX for rHPT, separately for those on dialysis and those with a functioning renal transplant.

**Methods:**

A systematic literature search was performed in electronic databases. Quality assessment was performed using the ﻿Quality In Prognosis Studies tool. Mean ioPTH values were calculated at different time points and correlated to the postoperative PTH levels within 1 month. Fixed-effect and random-effects models were performed to assess the mean ioPTH levels at 10 or 20 min after resection (T10 and T20). Stratified analyses were performed for patients on dialysis and those with a functioning renal transplant.

**Results:**

Of the 3087 records screened, 14 studies were included, including some 1177 patients; 1091 were on dialysis and 86 had a functioning kidney transplant. Risk of bias was moderate for most studies. For patients on dialysis, T10 and T20 mean ioPTH levels were 32.1 (95 per cent c.i. 24.3 to 39.9) pmol/l and 15.4 (95 per cent c.i. 7.8 to 22.9) pmol/l) in the random effects meta-analysis. Between individual studies, ioPTH ranged from 4.0–65.1 pmol/l at T10 and 8.6–25.7 pmol/l at T20. T10 and T20 ioPTH were 9.6 and 4.1 times the postoperative PTH—after T20 ioPTH stabilized in those on dialysis. In patients with a functioning renal transplant, ioPTH levels seemed to plateau after 10 min and measured 2.6 times the postoperative PTH.

**Conclusion:**

There is a strong correlation between ioPTH and early postoperative PTH levels, indicating that ioPTH is potentially a useful instrument during PTX in patients with rHPT. For patients on dialysis, at T20 ioPTH levels have stabilized and are approximately four times the postoperative PTH. Therefore, it is recommended to use ioPTH 20 min after resection in patients on dialysis, which might be longer than necessary for those with a kidney transplant.

## Teaser

Surgery for renal hyperparathyroidism is difficult—the surgeon must decide how much parathyroid tissue to remove and how much to leave behind. This systematic review and meta-analysis shows that intraoperative measurements of parathyroid hormone during parathyroidectomy for renal hyperparathyroidism can predict early parathyroid hormone levels and could therefore be useful.

## Introduction

Hyperparathyroidism (HPT) is common in chronic kidney disease (CKD), and associated with an increased risk of fractures, cardiovascular disease and death^1–6^. Renal HPT (rHPT) is treated medically, but if medical treatment cannot control the condition, surgical parathyroidectomy (PTX) reduces levels of parathyroid hormone (PTH), ameliorates rHPT and has decreased mortality rates in observational studies^7–14^. There are no specific guidelines for PTH levels after PTX, but Kidney Disease Improving Global Outcomes (KDIGO) and Kidney Disease Outcomes Quality Initiative (KDOQI) guidelines suggest PTH levels of 2 to 9 times times the upper normal limit for the assay, or in the range of 15–30 pmol/l in patients on dialysis, treated medically^15,16^. This is based on large observational data showing increased risk of adverse outcomes both with very low and very high levels of PTH^5,6^.

PTX is either performed as subtotal or total PTX with or without autotransplantation^9,10,17–19^. Both procedures can be combined with thymectomy. To help the surgeon decide how much parathyroid tissue to remove and how much to leave in the neck, several authors have used intraoperative measurement of PTH levels (ioPTH), to determine the extent of PTX^20–26^. However, no standard guideline or consensus exists regarding the timing of measurement of PTH during surgery, nor regarding whether absolute levels of PTH or levels relative to preoperative PTH levels should be used for decision-making^17^. The 2015 European Society of Endocrine Surgeons (ESES) consensus report states that the exact role of ioPTH in rHPT is undefined^17^. This is in contrast to ioPTH for primary hyperparathyroidism (pHPT), which has been demonstrated to enable focused surgery with less dissection, while maintaining excellent outcomes^27^. The most common protocol entails measuring PTH before incision and at 10 min after excision; a 50 per cent drop between these values indicates cure^27^.

Since there have been many reports on ioPTH in PTX for rHPT, but no consensus exists, the aim was to review the literature on ioPTH during PTX for rHPT systematically and appraise it critically to synthesize data and provide summary estimates. The main aim of this systematic review was to determine whether there is a correlation between ioPTH and levels of PTH at 1 day, 1 week or 1 month after surgery, and to determine the magnitude of this correlation. Other aims were to determine whether absolute ioPTH levels correlate better with postoperative levels of PTH, or if relative values (that is, percentage drop) are more useful to predict postoperative PTH. In addition, the optimal timing of ioPTH measurements, for example 10 or 20 min after resection, to offer the best prediction of postoperative PTH levels was examined. Since PTH kinetics could differ based on underlying rHPT aetiology, patients on dialysis and those with a functioning renal transplant were studied separately.

## Methods

This systematic review is reported according to the PRISMA statement^28^. Methods of the search, inclusion and exclusion criteria, quality assessment, and analyses were specified in advance in a protocol. No ethical approval was needed since data from previously published studies (in which ethical approval and/or informed consent were obtained by primary investigators) were retrieved and analysed.

### Search strategy

A systematic literature search was performed by one researcher (D.J.v.B.) on 22 November 2019, to identify articles that assessed ioPTH measurements to predict early postoperative PTH levels in patients undergoing PTX for rHPT. The search was performed in the electronic bibliographic databases of MEDLINE^®^ (PubMed), EMBASE^®^, Web of Science and Cochrane databases using the following search keywords and Boolean operators (‘Renal hyperparathyroidism’ OR ‘Chronic kidney disease’) AND ‘parathyroid surgery’ AND ‘intraoperative PTH’ and their corresponding synonyms. Database subject terms, such as Mesh terms (MEDLINE) and Emtree terms (EMBASE), were used as appropriate. The full search strings are reported in *Appendix S1*. There was no restriction for the year of publication of the studies and no other search filters were applied. All included articles were manually cross-referenced for additional relevant articles.

### Study selection

The results of the literature search were uploaded in the Rayyan^®^ database and duplicate cases were removed^29^. The titles and abstracts of the retrieved results were assessed in a blinded standardized manner for their relevance by two researchers (D.J.v.B., M.A.) independently. Inconsistencies were solved by consensus. The help of a third reviewer was not needed. Thereafter, the full texts of the records which were deemed potentially eligible were assessed. Reasons for exclusion at full-text screening were recorded.

### Eligibility criteria

Inclusion criteria were the reporting of ioPTH measurements during PTX for rHPT. rHPT was defined as active renal replacement therapy (haemodialysis or peritoneal dialysis), a (previous) kidney transplant, or CKD stage 3 or above (glomerular filtration rate (GFR) less than 60 ml/min/1.73^2^) without renal replacement therapy^15^. Patients with re-do PTX were included, but studies specifically focusing on ioPTH during surgery for parathyroid autograft recurrence in rHPT were excluded. To be included, studies had to report continuous PTH values or percentages of PTH decrease between 1 and 30 days after surgery. Randomized controlled trials, cohort studies, and case–control studies were eligible. Language was restricted to English, Swedish, German and Dutch. Exclusion criteria included the reporting of categorized or grouped outcomes based on cut-offs of PTH levels (without reporting continuous PTH data), absence of PTH levels between 1 and 31 days after PTX and/or lack of reporting of detailed ioPTH values at different time points. If studies investigated ioPTH in patients with pHPT and rHPT, and data of patients with rHPT could not be extracted separately the study was excluded. Also, original studies including fewer than five patients and articles for which no full text could be retrieved were excluded. Guidelines, literature reviews, case reports, editorials and commentaries were excluded.

If more than one publication of one study population was found only the most appropriate (that is, matching the review question) and with the most complete data was included.

### Risk-of-bias assessment

The risk of bias was assessed on a study level using the Quality In Prognosis Studies (QUIPS) tool for prognostic studies, which addresses six domains, including study participation, study attrition, prognostic factor measurement, outcome measurement, study confounding, and statistical analysis and reporting^30^. The tool was modified to fit this systematic review (*Appendix S2*). Study participation, prognostic factor measurement and outcome measurement were considered the most important domains and were therefore given the largest weight when assessing the final judgement of an individual study. The quality assessment was performed independently by two authors (D.J.v.B., S.F.) and disagreements were solved by discussion.

### Data extraction

Data extraction was performed by two authors (D.J.v.B., S.F.) using a standardized Microsoft Excel^®^ (Microsoft, Redmond, Washington, USA) extraction form; the form was established after conducting a test using representative studies. Relevant items were based on the CHARMS-PF (Critical Appraisal and Data Extraction for Systematic Reviews of Prediction Modelling Studies-Prognostic Factor) checklist^31,32^. Study characteristics (author, year, journal, country/location, study design (randomized controlled trial, cohort study), patient inclusion (single-/multicentre), data collection (prospective or retrospective), number of centres, number of patients, inclusion period, consecutive sample, inclusion and exclusion criteria), data regarding study population (age, sex, BMI, GFR, CKD stage, renal replacement therapy/dialysis, kidney transplant, duration of dialysis, vitamin D, calcimimetics), type of surgery (less than subtotal, subtotal, total PTX), parathyroid autotransplantation, thymectomy, preoperative laboratory values (PTH, calcium (total, ionized), phosphate), ioPTH measurements (time points, number of measurements, PTH values at each time point), assay and manufacturer (serum or plasma measurement, intact or whole PTH, generation of assay, coefficient of variation, calibration of assay, reference values, unit of measurement), PTH levels within the first postoperative month (time, measurement method) and reported potential confounders were extracted. No attempts were made to obtain unpublished data or to obtain raw data. Several studies only reported intra- and/or postoperative PTH levels within figures or graphs. These PTH values were extracted from the individual graphs by two authors (D.J.v.B., S.F.). In the case of differences in measurements, the average of the measurements was used.

### Statistical analysis

Biochemical values were converted to and reported as SI units. PTH levels were converted to pmol/l (pg/ml was multiplied by 0.106), calcium (total and ionized) to mmol/l (mg/dl was multiplied by 0.2495 (total calcium) or 0.25 (ionized calcium)), phosphate to mmol/l (mg/dl was multiplied by 0.3229) and creatinine to μmol/l (mg/dl was multiplied by 88.4)^15^.

Descriptive statistics were reported as mean(s.d.) or counts (percentages). Mean(s.d.) error of the mean was converted to mean(s.d.) based on the formula as reported in the Cochrane handbook^33^. Additionally, if studies reported values within two subgroups mean(s.d.), these were combined based on the formulae reported in the Cochrane handbook^33^. If studies reported non-normally distributed values, these were transformed to mean(s.d.) first. Data were transformed from non-normally distributed data (i.e. median and interquartile range or range) into mean(s.d.) based on formulae proposed by Wan and colleagues^34^. For studies only reporting individual patients’ data (IPD), group measures were calculated based on the provided IPD.

Aggregate data were calculated by summing individual studies’ group estimates which were weighted for the sample size for categorical variables and, in cases of continuous data (age, dialysis duration, PTH, total and ionized calcium and phosphate), also for the standard deviation^33^.

Aggregate pre-, intra- and postoperative PTH levels (pooled mean(s.d.)) were derived according to methods described before. Absolute PTH values, PTH levels as a ratio of the postoperative PTH level (that is, ioPTH value divided by the postoperative PTH value) and PTH levels as a percentage of the induction PTH levels value (that is, (ioPTH divided by the induction/preoperative PTH) times 100) were calculated and plotted. Mean(s.d.) of ioPTH values were determined based on the studies reporting at specific time points. All analyses were performed separately for patients on dialysis *versus* those with a functioning kidney transplant at the time of PTX, since renal function is markedly different between these groups, influencing the metabolism of PTH and hence the kinetics of drop of ioPTH. If studies did not explicitly state that patients had a kidney transplant these patients were analysed in the dialysis group.

As a pragmatic approach, both fixed-effect and random-effects meta-analyses were performed for ioPTH by using the generic inverse variance method, providing weighted means with subsequent 95 per cent confidence intervals of the mean. By applying the inverse-variance method, studies are weighted by the inverse of the variance of the PTH level (1 over the square of its standard error). Larger studies—which generally have smaller standard errors—receive more weight than smaller studies, which generally have larger standard errors. By applying the weights, the imprecision (uncertainty) of the pooled effect estimate is minimized^35^. Fixed-effect meta-analyses assume a common intervention effect and presume the absence of between-study heterogeneity. By contrast, random-effects meta-analyses expect differences in ioPTH levels between studies^35,36^. The *I^2^*—which describes the percentage of variability in effect estimates that is due to heterogeneity and not due to chance—was used to quantify the amount of heterogeneity between studies^37^. ioPTH levels at 10 and 20 min after resection (T10 and T20) were analysed, since these represent the most commonly used time points. Forest plots were used to illustrate graphically mean PTH values and subsequent 95 per cent confidence intervals at different time points of individual studies and meta-analyses^38,39^. To explore the type of underlying rHPT as a possible source of heterogeneity, the *I^2^* was compared between patients with and without a kidney transplant. Data were analysed using R, version 3.5.1 with ‘Metamean’ package (R Foundation for Statistical Computing, Vienna, Austria).

## Results

### Study selection

**Fig. 1:**
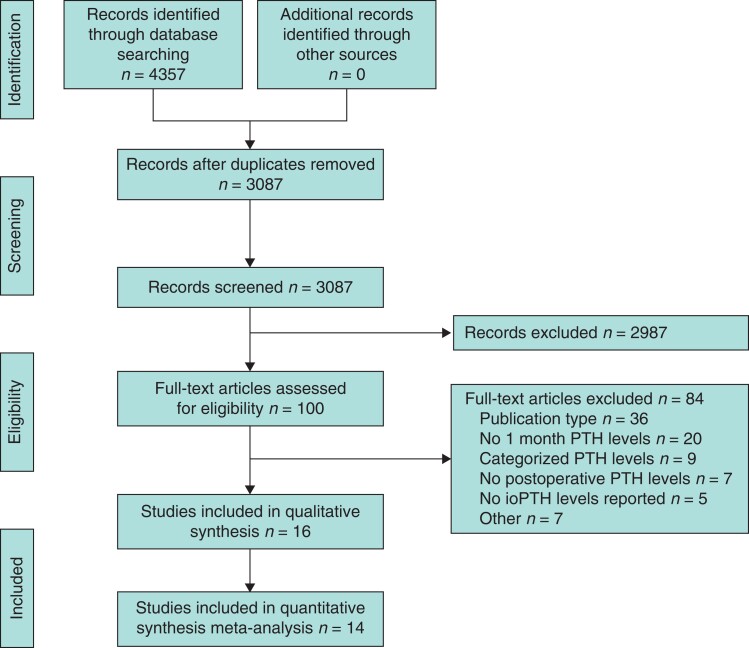
Flow chart of study selection PTH, parathyroid hormone; ioPTH, intraoperative parathyroid hormone.

The search yielded a total of 4357 records, of which 1240 were duplicates (*Fig. 1*). After removing the duplicates, 3087 records were screened of which 100 full texts were assessed for eligibility. In all, 32 studies were excluded because of reporting long-term outcomes and no short-term outcomes (20 studies), categorized outcomes after one month (9 studies) or lack of reporting of ioPTH values (3 studies) (*Appendix S3*). Sixteen studies were included after full-text review^21,40–53^. Since four studies included patients from two overlapping cohorts, the most recent cohorts, including the highest number of patients, were included^41,42,47,48^. A total of 14 papers were included for qualitative and quantitative analysis^21,40,42,44–47,49–53^.

### Study characteristics

Study characteristics of the 14 included studies are presented in *Table 1*. Most studies were performed in Europe (9 studies), three in Asia, one in the USA and one in Turkey. All studies were single-centre studies and most patients were included from the year 2000 onwards. Only one study was a randomized controlled trial^21^. Reported criteria for surgery varied widely between studies and included PTH levels, hypercalcaemia, hyperphosphataemia, symptoms refractory to medical treatment, enlarged parathyroid glands on imaging and/or KDIGO guideline criteria. Nine studies reported solely on patients on dialysis^21,40,43,45–47,50–52^, one study solely on patients with a functioning renal transplant^54^, three studies on both populations^42,49,53^ and in one study it was not explicitly reported^44^.

**Table 1:** Characteristics of included studies and patients

First author	No. of patients	Country	Study design	Data collection	Period of inclusion	Age at surgery*	Male
**Barczynski *et al*.^21^**	102	Poland	RCT	Prospective	12/2002–11/2003	38.1 (11.7)^†^	71 (70)
**Chou *et al*.^40^**	24	Taiwan	Cohort study	Retrospective	08/2002–12/2002	52 (11.8)^‡^	9 (38)
**Conzo *et al*.^46^**	35	Italy	Cohort study	Retrospective	01/2006–01/2009	52.0 (10.9)^‡^	18 (51)
**Echenique-Elizondo *et al*.^48^**	35	Spain	Cohort study	Prospective	01/2002–12/2006	52.7 (7.9)	16 (46)
**El-Husseini *et al*.^49^**	75	USA	Cohort study	Retrospective	03/2005–02/2015	47.1 (14.4)	36 (48)
**Kara *et al*.^50^**	42	Turkey	Cohort study	Retrospective	05/2006–07/2008	41.48 (1.9)^‡^	26 (62)
**Lorenz *et al*.^51^**	23	Germany	Cohort study	Retrospective	1997–2004	49 (10.3)	15 (65)
**Matsuoka *et al*.^52^**	44	Japan	Cohort study	Retrospective	—	53.4 (11.4)	21 (48)
**Müller-Stich *et al*.^53^**	26	Switzerland	Cohort study	Retrospective	10/1999–10/2004	n.r.	n.r.
**Seehofer *et al*.^41^**	153	Germany	Cohort study	Retrospective	10/1999–12/2003	50 (24.7)^‡^	86 (56)
**Triponez *et al*.^54^**	35	France	Cohort study	Retrospective	01/1998–12/2003	47 (11.0)	18 (51)
**Vulpio *et al*.^43^**	42	Italy	Cohort study	Retrospective	2007–2014	53.9 (14.8)	30 (71)
**Walgenbach and Junginger^44^**	40	Germany	Cohort study	Prospective	10/1999–05/2003	n.r.	n.r.
**Zhang *et al*.^45^**	501	China	Cohort study	Retrospective	04/2011–08/2015	45.9 (11.4)	277 (55)

Values in parentheses are percentages unless indicated otherwise; *values are mean(s.d.). ^†^Studies reported values within two subgroups which were combined based on the formulae reported in the Cochrane handbook.^33^ If studies reported non-normally distributed values, these were transformed to mean(s.d.) first. ^‡^One patient underwent two procedures. n.r., not reported.

### Patient characteristics

Characteristics of the patients in the 14 studies are reported in *Table 2*. Taken together, the 14 studies reported on a total of 1177 patients. Their mean age at surgery was 46.8 years, and 623 (56.1 per cent) were male. Some 1091 patients (92.7 per cent) were on dialysis and 86 (7.3 per cent) had a functioning kidney transplant at the time of PTX. Five studies, including 751 patients, reported the cause of underlying kidney disease^40,42,45,49,53^. Of these 751 patients, the majority (70.7 per cent, 531 patients) had glomerulonephritis as the underlying kidney disease^40,42,45,49,53^. Accumulated mean time on dialysis was 41.7 months and time between kidney transplant and PTX was 53.4 months. In those studies reporting on patients with a renal transplant, patients were reported to have a functioning transplant (*Table S1*)^42,49,53,54^.

**Table 2:** Patient characteristics and preoperative laboratory values

Study	No. in dialysis	Dialysis duration in months*	SPTX	TPTX	AT	TX	PTH (pmol/l)*	Ca total (mmol/l)*	P (mmol/l)*
**Barczynski *et al*.^21^**	102 (100)	88.6 (8.6)†	102 (100)	0	0	n.r.	158.7 (80.6)	2.55 (0.04)	n.r.
**Chou *et al*.^40^**	24 (100)	91.9 (32.3)§	0	24 (100)	24 (100)	n.r.	136.0 (44.7)§	2.67 (0.25)§	1.94 (0.48)§
**Conzo *et al*.^46^**	35 (100)	100.8 (50.4)§	35 (100)	0	22 (63)	n.r.	158.6 (85.7)§	2.54 (0.35)§	n.r.
**Echenique-Elizondo *et al*.^48^**	35 (100)	n.r.	0	35 (100)	35 (100)	35 (100)	138.0 ( 45.0)	n.r.	n.r.
**El-Husseini *et al*.^49^**	57 (76)	52.8 (28.8)	56 (75)	20 (27)‡	20 (27)	n.r.	117.5 (102.8)†	2.49 (0.22)†	1.74 (0.83)†
**Kara *et al*.^50^**	42 (100)	105.6 (46.7)†§	27 (64)	15 (36)	15 (36)	42 (100)	212.0 (78.4)†§	2.62 (0.16)† §	2.07 (0.55)†§
**Lorenz *et al*.^51^**	23 (100)	79.2 (44.4)	0	23 (100)	0	n.r.	152.6 (84.2)	2.50 (0.24)	n.r.
**Matsuoka *et al*.^52^**	44 (100)	154 (79)	0	44 (100)	44 (100)	44 (100)	86.9 (39.7)	n.r.	n.r.
**Müller-Stich *et al*.^53^**	17 (65)	n.r.	0	26 (100)	n.r.	24 (92)	143.0 (114.2)†¶	2.8	n.r.
**Seehofer *et al*.^41^**	129 (84)	94.8 (68.4) §	123^§^ (80)	13 (8)	<13 (8)**	n.r.	91.4 (60.3)†§	2.61 (0.27)†§	1.8 (0.61)†§
**Triponez *et al*.^54^**	0 (0)	n.r.	35 (100)	0	n.r.	35 (100)	32.4 (36.0)	2.79 (0.25)	n.r.
**Vulpio *et al*.^43^**	42 (100)	56.4 (30)	15 (36)	27 (64)	0	42 (100)	131.0 (52.7)†	2.62 (0.37)†	1.77 (0.75)†
**Walgenbach and Junginger^44^**	n.r.	n.r.	0	40 (100)	33 (83)	40 (100)	99.5 (54.6)†¶††	n.r.	n.r.
**Zhang *et al*.^45^**	501 (100)	90 (40.1) ¶	0	501 (100)	501 (100)	0	232.8 (113.1) ¶	2.54 (0.22)¶	2.16 (0.52) ¶

Values in parentheses are percentages unless indicated otherwise; *values are mean(s.d.). Parathyroid hormone (PTH) levels are reported as preoperative PTH levels. If no preoperative PTH levels were reported, induction PTH levels are presented (El-Husseini *et al*.^49^, Müller-Stich *et al*.^47^, Vulpio *et al*.^51^, Zhang *et al*.^53^). †Studies reported values within two subgroups which were combined based on the formulae reported in the Cochrane handbook^33^. If studies reported non-normally distributed values, these were transformed to mean(s.d.) first. ‡One patient underwent two procedures. §Mean(s.e.m.) was converted to mean(s.d.) based on the formula reported in the Cochrane handbook^33^. ¶Data were transformed from non-normally distributed data (median + interquartile range/range) into mean(s.d.) based on the formulae proposed by Wan *et al*.^34^. #In the remaining 17 patients—with recurrent disease—the respective hyperfunctioning tissue was removed. **Not clearly reported, but fewer than 13 patients. ††Data were extracted from figures. AT, autotransplantation; Ca, calcium; P, phosphate; SPTX, subtotal parathyroidectomy; TPTX, total parathyroidectomy; TX, thymectomy; n.r., not reported.

Subtotal PTX and total PTX with or without autotransplantation were performed in 375 (31.9 per cent) and 802 patients (68.1 per cent). Two hundred eighty-seven patients underwent simultaneous thymectomy together with PTX. In the 905 patients on dialysis with available data, subtotal and total PTX with or without autotransplantation were performed in 179 (19.8 per cent) and 726 patients (80.0 per cent) respectively. Of those 44 patients with a functioning kidney transplant and available data, 35 underwent subtotal PTX and nine total PTX. For the remaining 228 patients the studies did not report surgical procedures stratified for dialysis or transplanted patients, but the majority underwent subtotal PTX^42,49^.

Mean levels of preoperative PTH, total calcium and phosphate were 9.0 pmol/l, 2.56 mmol/l and 2.04 mmol/l respectively. In patients on dialysis and those with a kidney transplant, preoperative or induction mean PTH levels were 170.7 and 54.4 pmol/l respectively.

In four studies ioPTH measurements altered surgical strategies^21,40,43,51^, in the other 10 studies patients underwent planned surgical procedures and ioPTH levels did not influence the extent of parathyroidectomy or its potential was not reported^42,44–47,49,50,52–54^. In 31 patients (2.6 per cent) a change in surgical management was reported^21,40,43,51^; in two^40^ and six^43^ patients the extended surgical exploration did not yield additional parathyroid tissue.

### Quality assessment

Quality assessment of the included studies is shown in *Table 3*. Three studies had a low risk of bias^43,46,54^, six a moderate risk of bias^21,40,45,49,50,53^ and five a high risk of bias^42,44,47,51,52^. Three studies described the ioPTH and postoperative PTH assay in detail (e.g. type of assay, coefficient of variation, manufacturer, reference range), only five reported a coefficient of variation and none reported whether measurements in the laboratories were calibrated and validated (*Table S2*). Potential confounders were poorly reported and definitions for confounders were rarely given. For example, only two studies described a definition of how kidney function was estimated^53,54^.

**Table 3:** Risk of bias

Study	Study participation	Study attrition	Prognostic factor measurement	Outcome measurement	Confounders	Statistical analysis	Overall risk of bias
**Barczynski *et al*.^21^**	L	L	M	M	H	M	M
**Chou *et al*.^40^**	M	L	M	M	H	M	M
**Conzo *et al*.^46^**	L	L	L	L	H	M	L
**Echenique-Elizondo *et al*.^48^**	L	L	M	H	H	M	H
**El-Husseini *et al*.^49^**	L	M	M	L	H	M	M
**Kara *et al*.^50^**	L	L	M	M	H	M	M
**Lorenz *et al*.^50^**	H	L	H	H	H	L	H
**Matsuoka *et al*.^52^**	M	L	M	H	H	H	H
**Müller-Stich *et al*.^53^**	M	L	M	L	M	M	M
**Seehofer *et al*.^41^**	H	L	M	L	H	M	H
**Triponez *et al*.^54^**	L	L	M	L	M	M	L
**Vulpio *et al*.^43^**	L	L	L	L	H	M	L
**Walgenbach and Junginger^44^**	H	M	L	L	H	M	H
**Zhang *et al*.^45^**	L	H	M	L	H	M	M

L, low risk of bias; M, moderate risk of bias; H, high risk of bias.

### Timing of intraoperative parathyroid hormone assays

The timing of ioPTH and the used assays are summarized in *Table 4* and *Table S2*. One study compared two PTH assays^40^. Eleven studies reported an induction PTH value^21,40,43,45–47,49,51–54^, three used either a preoperative value^44,50^ or value after skin incision and exposure of the thyroid gland but before preparation of the parathyroid glands^42^ as baseline value. Most studies (11 studies) measured ioPTH 10 min after resection of the last parathyroid gland followed by seven studies reporting ioPTH after 20 min. In the 13 studies reporting on patients on dialysis, 10 and six reported ioPTH at T10 and T20, respectively. For the four studies including patients with a functioning renal transplant, three and two reported at T10 and T20 respectively.

**Table 4:** Absolute intraoperative parathyroid hormone levels

Study	Preop.	T0 induction	T5	T10	T15	T20	T30	Postop.	PTH
**Patients on dialysis**									
Barczynski *et al*.^21^		158.7 (80.6)*		33.7 (15.6)*				1 Month	3.0 (0.8)*
Chou *et al*.^40^	136.0 (44.7)†	104.7 (55.7)†		18.7 (21.2)†			10.1 (12.1)†	1 Week	2.3 (2.5)†
Conzo *et al*.^46^	158.6 (85.7)*†	136.2 (136.6)*†		56.6 (35.1)*†		15.3 (10.3)*†		1 Day	5.0 (5.1)*†
Echenique-Elizondo *et al*.^48^	138.0 (45.0)	142.4 (53.6)‡§	93.5 (36.9)‡§	65.1 (20.8)	14.3 (9.2)‡§	8.6 (2.6)‡§	3.6 (0.0)‡§	1 Day	0
El-Husseini *et al*.^49^		161.2 (99.8)‡		16.4 (13.4)‡		11.5 (7.6)‡		1 Week	2.4 (2.9)‡§
Kara *et al*.^50^	212.0 (78.4)*†				21.1 (17.4)*†			1 Week	11.5 (27.1)*§
Lorenz *et al*.^51^	152.6 (84.2)	151.6 (90.6)		12.8 (13.4)		12.1 (14.8)	1.2 (14.3)	1–3 Days	13.6 (28.8)
Matsuoka *et al*.^52^	86.9 (39.7)	78.8	6.2	4.0	2.9		1.7	1 Day	1.4 (1.2)
Müller-Stich *et al*.^53^		172.6 (118.9)*‡		22.1 (16.0)*‡				1–10 Days	5.4 (5.4)*‡
Seehofer *et al*.^41^	88.6 (55.3)*†				17.7 (17.7)*†			1–3 Days	4.5 (10.3)*†
Vulpio *et al*.^43^		131.0 (52.7)		34.7 (21.6)*‡§		18.8 (7.9)*‡§	15.1 (6.2)*‡§	1 Week	2.8 (3.3)*‡§
Walgenbach and Junginger^44^	99.5 (54.6)		21.5 (17.5)*‡§					1 Day	4.9 (10.3)*‡§
Zhang *et al*.^45^		232.8 (113.1)*‡		32.9 (16.0)*‡§		25.7 (13.1)*‡§		1 Day	6.9 (24.0)*‡
**Patients with a functioning kidney transplant**									
El-Husseini *et al*.^49^		30.2 (19.0)‡		4.9 (4.0)‡		3.6 (1.9)‡		1 Week	7.9 (8.9)‡§
Müller-Stich *et al*.^53^		71.0 (61.8)*‡		3.6 (2.1)*‡				1–10 Days	3.7 (4.6)*‡
Seehofer *et al*.^41^	53.5 (35.3)*†				9.0 (4.7)*†			1–3 Days	3.7 (9.3)*‡
Triponez *et al*.^54^		32.4 (36.0)	14.2 (8.8)‡§	11.9 (7.7)‡§		9.6 (6.7)‡§	8.6 (5.7)‡§	1 Day	3.0 (1.5)

All values are reported as mean(s.d.); Matsuoka *et al*.^52^ did not report a measure of dispersion. All studies, except for Conzo *et al*.^46^, reported parathyroid hormone (PTH) levels as pg/ml; these were converted into pmol/l (pg/ml times 0.106)^15^. *Studies reported values within two subgroups which were combined based on the formulae reported in the Cochrane handbook^33^. If studies reported non-normally distributed values, these were transformed to mean(s.d.) first. †Mean(s.e.m.) was converted to mean(s.d.) based on the formula reported in the Cochrane handbook^33^. ‡Data were transformed from non-normally distributed data (i.e., mean(i.q.r.)/range) into mean(s.d.) based on the formulas proposed by Wan *et al*.^34^. ^§^Data were extracted from figures. Preop., preoperative; Postop., postoperative; T, time point.

In addition, Lorenz and colleagues^51^ reported ioPTH values after the resection of each parathyroid gland and Walgenbach and Junginger^44^ reported ioPTH levels 5 min after the exploration and resection of the parathyroid glands on the right side and 5 min after the left side. The only randomized study, by Barczyński and co-workers^21^, allocated patients to the measurement of either six (preoperative, pre-excision, after 5, 10, 20 and 60 min) or two (preoperative and after 10 min) consecutive samples.

In the 14 studies included in this review, levels of PTH were reported on postoperative day 1^44–47,52,54^ (6 studies), within the first week^42,51,53^ (3 studies), after 1 week^40,43,49,50^ (4 studies) or after 1 month^21^ (1 study).

### Intraoperative parathyroid hormone values in patients on dialysis

**Fig. 2:**
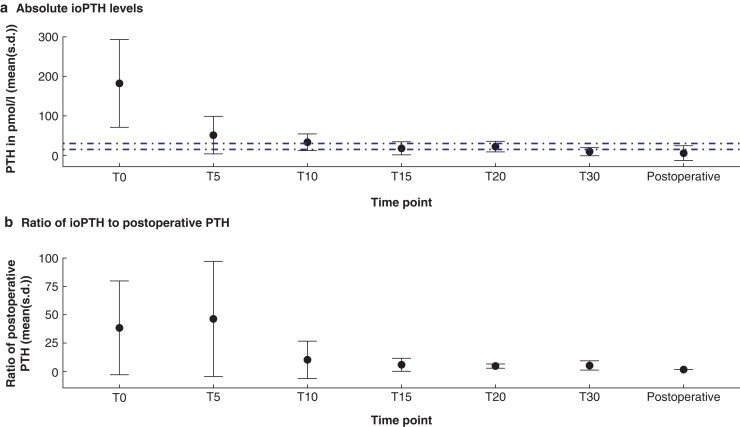
Aggregate parathyroid hormone levels at different pre-, intra- and postoperative time points in patients on dialysis

*Fig. 2* shows the course of the pooled mean(s.d.) of the absolute ioPTH levels in patients on dialysis; individual studies’ ioPTH levels at different time points are reported in *Table 4*. Mean(s.d.) induction PTH (103.5(111.1) pmol/l) was well above the KDIGO recommended range. Mean(s.d.) values decreased to within the KDIGO reference range from T10 (33.2(21.1) pmol/l) until T20 (22.2(13.5) pmol/l) and were below the reference range at the first follow-up (5.6(18.6) pmol/l). Between individual studies, ioPTH ranged from 4.0–65.1 pmol/l at T10 and 8.6–25.7 pmol/l at T20. Pooled standard deviations could not be calculated for the study by Matsuoka and colleagues^47^ since no measures of dispersion were reported and could not be extracted. This study was therefore excluded from these analyses.

**a** Absolute intraoperative parathyroid hormone (ioPTH) levels. The dashed lines indicate the recommended KDOQI guidelines target range of 15.9–31.8 pmol/l. **b** Ratio of ioPTH to postoperative parathyroid hormone (PTH). Mean and standard deviations are derived by formulae reported in the Cochrane handbook^33^. Number of studies and patients available for analysis at each time point: T0 induction: 9 studies, 1047 patients; T5: 2 studies, 75 patients; T10: 9 studies, 954 patients; T15: 3 studies, 206 patients; T20: 6 studies, 693 patients; T30: 4 studies, 124 patients; postoperative: 9 studies, 1047 patients.

Pooled mean pre-induction PTH levels were 38.1 times the first postoperative PTH levels (*Fig. 2* and *Table 5*). From T10 onwards—9.6 times the first postoperative PTH (range between individual studies 0.9–65.1)—weighted mean PTH levels stabilized. At T15, T20 and T30, weighted mean PTH levels were 5.2 (range 1.8–14.3), 4.1 (range 0.9–8.6) and 4.7 (range 0.1–5.5) times the first postoperative PTH level, respectively.

**Table 5:** Ratio of intraoperative parathyroid hormone to postoperative parathyroid hormone

Study	T0 induction	T0 surgery*	T5	T10	T15	T20	T30
**Patients on dialysis**							
Barczynski *et al*.^21^	52.3 (101.3)†			11.1 (19.)†			
Chou *et al*.^40^	44.9 (22.4)‡	21.7 (19.1)‡		8.0 (8.5)‡			4.4 (4.8)‡
Conzo *et al*.^46^	27.1 (26.9)‡†			11.3 (6.9)†‡		3.0 (2.0)†‡	
Echenique-Elizondo *et al*.^48^	142.4 (53.6)§¶	152.3 (59.7)§¶	93.5 (36.9)§¶	65.1 (20.8)	14.3 (9.2)§¶	8.6 (2.0)§¶	3.6 (0.0)§¶
El-Husseini *et al*.^49^	66.1 (34.4)§			6.7 (4.6)§		4.7 (2.6)§	
Kara *et al*.^50^	1.8 (2.9)†‡				1.8 (0.6)†‡		
Lorenz *et al*.^51^	11.1 (3.1)			0.9 (0.5)		0.9 (0.5)	0.1 (0.1)
Matsuoka *et al*.^52^	55.2	7.8	4.4	2.8	2.0		1.2
Müller-Stich *et al*.^53^	31.9 (22.1)†§			4.1 (3.0)†§			
Seehofer *et al*.^41^	19.7 (5.4)†‡	20.1 (6.3)†‡			3.9 (1.7)†‡		
Vulpio *et al*.^43^	47.3 (15.9)	47.9 (17.5)		12.5 (6.5)†§¶		6.8 (2.4)†§¶	5.5 (1.9)†§¶
Walgenbach and Junginger^44^	20.1		4.4 (1.7)§†¶				
Zhang *et al*.^45^	33.8 (4.7)†§			4.8 (0.7)†§		3.7 (0.5)†§	
**Patients with a functioning kidney transplant**							
El-Husseini *et al*.^49^	3.8 (2.1)§			0.6 (0.5)§		0.5 (0.2)§	
Müller-Stich *et al*.^53^	19.3 (13.4)†§			1.0 (0.5)†§			
Seehofer *et al*.^41^	14.4 (3.8)†‡	15.1 (4.0)†‡			2.4 (0.5)†‡		
Triponez *et al*.^54^	21.6 (32.1)		4.8 (5.9)§¶	4.0 (5.2)§¶		3.2 (4.5)§¶	2.9 (3.9)§¶

All values are reported as mean(s.d.); Matsuoka *et al.*^52^ did not report a measure of dispersion. Echenique-Elizondo *et al*.^48^ had a mean postoperative parathyroid hormone (PTH) of 0 pmol/l, this was arbitrarily set at 1.0 pmol/l to calculate the ratio. *T0 surgery levels were reported, before resection of last parathyroid gland (Conzo *et al*.^46^), at cut time (Echenique-Elizondo *et al*.^48^), immediately after removal of the last gland (Matsuoka *et al*.^52^), after skin incision and exposure of the thyroid, but before preparation of the parathyroid glands (Seehofer *et al*.^41^), after manipulation (Triponez *et al*.^54^). †Studies reported values within two subgroups which were combined based on the formulae reported in the Cochrane handbook^33^. If studies reported non-normally distributed values, these were transformed to mean(s.d.) first. ‡Mean(s.e.m.) was converted to mean(s.d.) based on the formula reported in the Cochrane handbook^33^. §Data were transformed from non-normally distributed data (median + interquartile range/range) into mean(s.d.) based on the formulae proposed by Wan *et al.*^34^. ^¶^Data were extracted from figures. T, time point.

Intraoperative and postoperative PTH levels, expressed as percentage of induction PTH levels, are shown in *Table S3*. On average, ioPTH at T10, T20 and postoperative were 17.2 (range 5.0–41.6), 10.6 (range 6.0–14.4) and 3.1 (range 0.7–9.0) per cent of the induction PTH, indicating a drop of 82.8, 89.4 and 96.9 per cent at these respective time points.

### Intraoperative parathyroid hormone values in patients with a functioning renal transplant

The course of the pooled mean(s.d.) of the absolute ioPTH levels in patients on dialysis is shown in *Fig. S1*. In patients with a functioning kidney transplant induction mean(s.d.) PTH levels were approximately half of the values reported in patients on dialysis (54.4(42.6) *versus* 103.5(111.1) pmol/l respectively) (*Table 4*). At T10, T20 and follow-up these decreased to 8.8(7.3) (range between individual studies 3.6–11.9), 7.6(6.2) (range 3.6–9.6) and 4.3(6.8) (range 3.0–7.9) pmol/l respectively.

During induction, PTH levels were 15.5 times (range 3.8–21.6) the postoperative PTH (*Figure S1* and *Table 5*). Levels were 2.6 (range 0.6–4.0) and 2.3 (range 0.5–3.2) times the postoperative PTH levels at T10 and T20 respectively. In terms of percentage, ioPTH at T10, T20 and postoperative were 16.3 (range 5.0–18.5), 13.9 (range 11.9–14.9) and 9.9 (range 4.6–26.1) per cent of induction PTH levels (*Table S3*), indicating of a decrease of 83.7, 86.1 and 90.1 per cent respectively.

### Meta-analyses of intraoperative parathyroid hormone levels in patients on dialysis

**Fig. 3:**
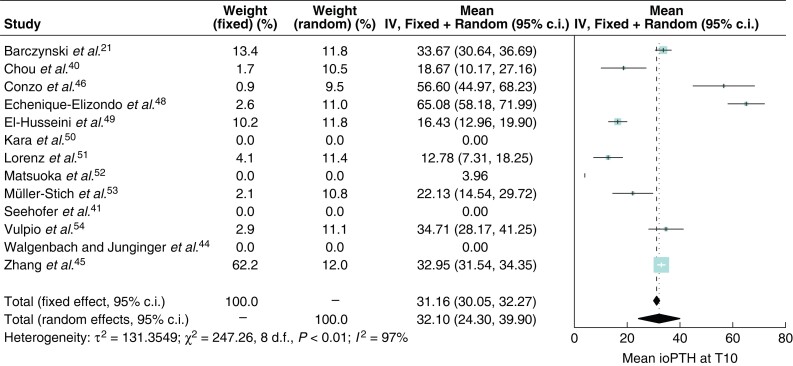
Forest plot of intraoperative parathyroid hormone levels at T10 in patients on dialysis ioPTH, intraoperative parathyroid hormone; IV, inverse variance; T, time.

**Fig. 4:**
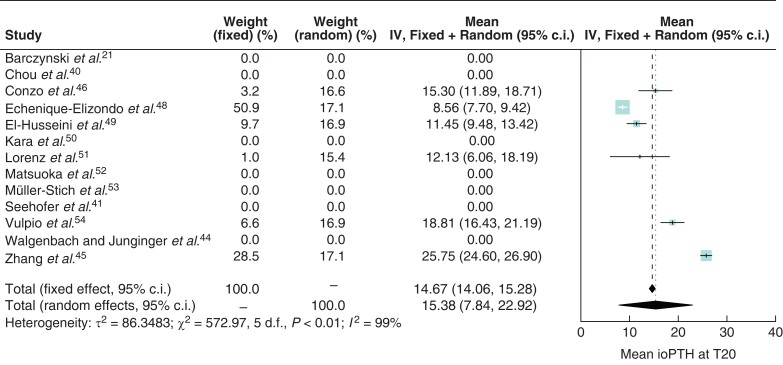
Forest plot of intraoperative parathyroid hormone levels at T20 in patients on dialysis ioPTH, intraoperative parathyroid hormone; IV, inverse variance; T, time.

*Figs. 3* and *4* show forest plots of ioPTH levels at T10 and T20 in patients on dialysis. The *I^2^* at T10 and T20 were 97 and 99 per cent respectively. The forest plots show a wide scatter of effect estimates with little overlap in confidence intervals between studies. In the random-effects model, at T10 mean ioPTH levels were 32.1 (95 per cent c.i. 24.3 to 39.9)  pmol/l and at T20 15.4 (95 per cent c.i. 7.8–22.9) pmol/l. Point estimates were similar between the fixed-effect and random-effects meta-analyses, however, as expected the 95 per cent confidence intervals of the fixed-effect models were unreasonably narrow since the fixed-effect model assumes the absence of between-study heterogeneity.

### Meta-analyses of intraoperative parathyroid hormone levels in patients with a functioning renal transplant

The meta-analyses of patients with a functioning kidney transplant at T10 and T20 are shown in *Figs. S2* and *S3*. In the random-effects meta-analyses, at T10 mean ioPTH levels were 6.7 (95 per cent c.i. 2.3 to 11.1) pmol/l and at T20 6.5 (95 per cent c.i. 6.7 to 12.4) pmol/l. The *I^2^* at these time points were 93 and 96 per cent respectively.

### Reporting and analysis of potential factors influencing the use and interpretation of intraoperative parathyroid hormone

As reported in *Table 2*, most studies reported on age at surgery (12 studies), sex (12 studies) and time on dialysis (10 of 13 studies). The time from renal transplant to PTX was reported in two out of four studies. Renal function (GFR or creatinine) at the time of surgery was reported in only five studies, of which four described it in patients with a kidney transplant^42,45,49,53,54^.

Some studies performed subgroup analyses for patients on dialysis *versus* those with a functioning kidney transplant^42,49,53^, subtotal PTX *versus* total PTX (with or without autotransplantation)^43,49^, total PTX *versus* total PTX plus autotransplantation^46^, GFR less than 60, and 60 or greater than 60 ml/min^54^, four or less than four observed parathyroid glands^44^ and type of assay^40^. None of the included studies performed multivariable analysis.

## Discussion

The systematic search of 3086 records identified 14 full-text studies reporting on ioPTH in PTX for rHPT. Most studies were of moderate methodological quality. A total of 1177 patients were included in these studies, of whom 1091 were on dialysis and 86 had a functioning renal transplant at the time of PTX. In patients on dialysis, the pooled ratios between ioPTH at 10 and 20 min and immediate postoperative PTH levels—determined within the first postoperative month—were approximately 10 and 4. This ratio differed between studies, ranging from 0.9 to 65.1 and 0.9 to 8.6. All studies reported a significant drop of ioPTH compared with preoperative PTH. On average, postoperative PTH levels were one-tenth of ioPTH at 10 min, one-fifth of ioPTH at 15 min, and one-quarter of ioPTH at 20 min in patients on dialysis. After 20 min, ioPTH did not decrease further in those on dialysis. In patients with a renal transplant, T10 and T20 ioPTH levels were 2.6 (range individual studies 0.6–4.0) and 2.3 (range individual studies 0.5–3.2) times the postoperative PTH levels, however, the small number of patients in this subgroup limits the interpretation of these data. Thus, this systematic review indicates that there is a clear correlation between ioPTH and early postoperative PTH in patients undergoing PTX for rHPT. It is suggested that ioPTH 20 min after resection is used for those on dialysis, however, for those with a functioning transplant 20 min after resection might be longer than necessary; additional data are needed for this subgroup.

The present study focused on short-term PTH levels, but the long-term stability of PTH beyond the immediate postoperative period was not investigated. Data on this topic are relatively scarce. One large cohort study using administrative data including 1165 patients undergoing PTX with postoperative PTH data observed stable PTH levels during the first year after PTX^7^. However, PTH levels were analysed monthly at a group level, indicating that PTH was not assessed longitudinally within individual patients. In a randomized controlled trial comparing total PTX *versus* total PTX plus autotransplantation with 3 years of follow-up, mean PTH levels remained relatively stable within both groups, although standard deviations changed over time and the mean PTH levels differed significantly between the two groups after 3 years^55^. Hence, for ioPTH to be useful, the endocrine surgeon and the nephrologist need not only to know whether ioPTH correlates with PTH at 1 day or 1 month, but also whether PTH levels remain stable after surgery, or if they will rise again after surgery. More observational research is needed on this topic to understand the long-term stability of PTH levels and to identify factors associated with unstable PTH levels.

The lack of clear evidence for an optimal level of PTH after PTX additionally hampers the concept of ioPTH monitoring. Renal transplantation, which generally offers the best outcomes for patients with renal failure, will dramatically impact rHPT by generally lowering levels of PTH^56^. Very low levels of PTH have been associated with poor outcomes, and it is possible that different patients with renal failure have different optimal levels of PTH after PTX^5,6,57^. For instance, PTX seems to reduce the risk of fractures more in women than in men—it is possible that patients with a higher risk of fractures need lower levels of PTH, whereas patients with cardiovascular risk need higher levels of PTH for optimal outcomes^58^. Future, large-scale observational studies, using levels of PTH after PTX to predict long-term outcomes, such as fractures, cardiovascular disease and death, are needed, to give advice on optimal PTH levels in individual patients.

There has been a heated debate among endocrine surgeons as to which procedure—subtotal or total PTX—offers the best long-term outcomes for patients with rHPT^10,17,19^. In the light of an increased availability of kidney transplants, a patient-tailored extent of PTX taking patient, disease and future kidney transplant prospects into account is probably needed^59^. Since rHPT is an incurable disease, the probable question is not how much parathyroid tissue to remove, but how much to leave behind in order to tailor the extent of surgery to the individual patient’s needs^18^. ioPTH, as evidenced by this systematic review, has the potential to aid the surgeon in estimating postoperative levels of PTH, since there was a strong correlation between ioPTH and postoperative levels of PTH. On average, within the first month, levels of PTH were approximately 25 per cent of ioPTH at 20 min in patients on dialysis and for those with a functioning transplant almost 40 per cent of ioPTH at 10 min. Nevertheless, some surgeons propose single- or double-gland resections for rHPT. Almost all patients studied in this analysis underwent subtotal or total PTX. Therefore, the observed results might not necessarily apply to patients undergoing lesser resections.

Besides the amount of parathyroid tissue to leave behind, the vascularization of the parathyroid glands should be taken into account^18^. Further adjuncts that might make parathyroid surgery for rHPT more precise include detection with autofluorescence and parathyroid angiography using indocyanine green^60,61^. However, evaluating the usefulness of these adjuncts, with or without ioPTH, was beyond the scope of this review. Future studies should preferably investigate the combined use of ioPTH with parathyroid angiography. Both techniques might lead to an accurate estimation of cumulative postoperative PTH levels as well as localization of (ectopic) parathyroid glands and estimation of the individual parathyroid gland function.

It is not uncommon for patients with rHPT to have supernumerary and/or ectopic parathyroid glands^62,63^. Finding all parathyroid glands can be challenging during PTX. In pHPT, studies indicate that preoperative localization, with ultrasonography, technetium-99m (^99m^Tc) sestamibi scintigraphy with or without single-photon emission CT, (4D) CT, MRI or PET-CT, aids the surgeon in finding and removing the diseased gland(s). However, in rHPT, preoperative localization has been less useful and thus the ESES consensus reports states that preoperative localization should be restricted to cervical ultrasonography, thereby underscoring the importance of conventional bilateral exploration^17^. A single-centre study, including 20 patients undergoing ultrasonography, ^99m^Tc sestamibi, CT and intraoperative angiography with indocyanine green observed sensitivities of 81.2, 62.3, 85.7 and 91.1 per cent respectively^60^. Preoperative localization was not taken into account in the present study.

The KDIGO recommendation range is for those on dialysis and not necessarily for those with a functioning transplant. Nevertheless, HPT is a common problem in those after transplantation, since 43 per cent suffer from HPT within 2 years after successful kidney transplantation and HPT negatively affects graft survival^56^. Therefore, stratified analyses were performed between those on dialysis and those with a functioning kidney transplant. Due to differences in kidney function, these two groups would be expected to have very different intrinsic kinetics of PTH metabolism. Preoperative PTH levels were lower in patients with a functioning kidney transplant. As observed in the present study, the transplanted group plateaued at 10 min. Although the number of patients with a transplant was relatively low, one could argue that using PTH levels 20 min after resection might be longer than necessary for those with a kidney transplant. In patients with sporadic primary HPT the half-life of PTH is approximately 3 min^64^. In a review including 19 studies studying patients with single-gland HPT, a 75 per cent decrease of ioPTH was observed 10 min after resection in all studies and ioPTH seemed to plateau thereafter^65^. Future studies should assess whether PTH kinetics in those with a functioning kidney transplant behave similarly to those with sporadic primary HPT.

The major strength of the present study is the extensive and comprehensive literature search in multiple databases yielding over 3000 articles. The methodological quality of the included articles was critically assessed and appraised according to predefined quality criteria tailored to the research topic. By applying methods for handling unreported mean or variability summary statistics, the number of included studies was maximized^66^. Despite techniques to obtain summary statistics, nine studies were excluded because of reporting categorized outcomes based on cut-off values to indicate cure *versus* no cure. Multiple analyses—including absolute PTH levels with subsequent random-effects and fixed-effect meta-analysis, ratio of postoperative PTH and percentage of decrease—were performed to investigate the usefulness of ioPTH in multiple respects. Furthermore, stratified analyses were performed for patients on dialysis and for patients with a functioning renal transplant, since induction PTH and ioPTH levels were generally lower in patients with a functioning transplant.

The main limitation of the present study is the lack of IPD. PTH levels were reported differently among studies and in some studies data had to be extracted from figures. With IPD, and data on type and variation of measurement of PTH (that is, coefficient of variation), a multivariable analysis, meta-regression or IPD meta-analysis (IPD-MA) could be performed, yielding information on individual predictive value of ioPTH on postoperative PTH, taking GFR, age, sex and other potential confounders or effect modifiers of ioPTH kinetics into account. Other statistical and clinical advantages of IPD-MA over aggregate data meta-analysis include the verification of originally published data including standardized statistical analysis, use of same measurement units and subsequent measures of dispersion, transformation of skewed data, opportunity to account for missing data and possibility of subgroup analyses^67^. In addition, the majority of studies had a moderate or high risk of bias, mainly due to the lack of detailed information regarding the ioPTH protocol or confounders. The studies included in the review were heterogeneous in terms of design ((randomized controlled) trial *versus* observational and prospective *versus* retrospective), study cohorts including mix of surgical procedures, and endpoints. Significant heterogeneity existed between studies—the *I^2^* at T10 and T20 indicate that over 90 per cent is caused by true study heterogeneity due to real study differences and only up to 10 per cent is caused by chance. The heterogeneity is most likely due to different surgical indications, access to endocrine surgeons and distributions of confounders between studies. In addition, missing data were observed in several studies, but most studies did not report on the presence or absence of missing data. No analyses were performed to assess potential publication bias.

IoPTH can be used to predict early postoperative PTH levels after PTX for rHPT and could therefore potentially be a useful tool. For patients on dialysis, ioPTH levels drop after 10, 15 and 20 min, whereafter they seem to plateau; at 20 min, levels are on average four times the levels at 1 day to 1 month. In patients with a functioning transplant, PTH seems to plateau after 10 min, but more data are needed for this subgroup considering the low number of patients. IPD (meta-)analysis would enable one to account for confounding factors to enable more precise estimations of postoperative PTH levels to tailor the extent of PTX for the individual patient with rHPT. In addition, the lack of clear evidence for an optimal level of PTH after PTX hampers the concept of ioPTH monitoring to enable tailored PTX. More observational research is needed on this topic to understand the long-term stability of PTH levels.

## Supplementary Material

zrab151_Supplementary_Data

## Data Availability

Data available on request. The data underlying this article will be shared on reasonable request to the corresponding author.
