# Supplementary material for: Use of intraoperative parathyroid hormone measurements during parathyroidectomy to predict postoperative parathyroid hormone levels in patients with renal hyperparathyroidism: meta-analysis
Source: BJS Open. 2022 Feb 15;6(1):zrab151. doi: 10.1093/bjsopen/zrab151 (PMC8855528; doi:10.1093/bjsopen/zrab151)
Supplement: zrab151_Supplementary_Data [file zrab151_supplementary_data.zip › Supplementary_Appendices.docx]

## *Appendix S1: Search terms and results*

### Pubmed

|  |  |  |
| --- | --- | --- |
| #1 | ((((((((((Renal hyperparathyroidism[Title/Abstract]) OR Renal HPT[Title/Abstract]) OR rHPT[Title/Abstract]) OR Secondary hyperparathyroidism[Title/Abstract]) OR Secondary HPT[Title/Abstract]) OR sHPT[Title/Abstract]) OR 2HPT[Title/Abstract]) OR Tertiary hyperparathyroidism[Title/Abstract]) OR Tertiary HPT[Title/Abstract]) OR 3HPT[Title/Abstract]) OR hyperparathyroidism, secondary[MeSH Terms] | 11779 |
| #2 | ((((((((((((((((((Chronic kidney disease[Title/Abstract]) OR Chronic kidney insufficiency[Title/Abstract]) OR CKD[Title/Abstract]) OR End stage kidney disease[Title/Abstract]) OR End-stage kidney disease[Title/Abstract]) OR End stage renal disease[Title/Abstract]) OR End-stage renal disease[Title/Abstract]) OR ESRD[Title/Abstract]) OR Renal insufficiency[Title/Abstract]) OR Dialysis[Title/Abstract]) OR Hemodialysis[Title/Abstract]) OR Haemodialysis[Title/Abstract]) OR Renal replacement therapy[Title/Abstract]) OR Kidney transplant[Title/Abstract]) OR Kidney transplantation[Title/Abstract]) OR Renal transplant[Title/Abstract]) OR Renal transplantation[Title/Abstract]) OR renal insufficiency, chronic[MeSH Terms]) | 306829 |
| #3 | ((((Parathyroidectomy[Title/Abstract]) OR Resection[Title/Abstract]) OR Surgery[Title/Abstract]) OR PTx[Title/Abstract]) OR parathyroidectomy[MeSH Terms] | 1365463 |
| #4 | ((((((((((((((((((((ioPTH[Title/Abstract]) OR intraoperative PTH[Title/Abstract]) OR intra-operative PTH[Title/Abstract]) OR intraoperative parathyroid hormone[Title/Abstract]) OR intra-operative parathyroid hormone[Title/Abstract]) OR intraoperative parathormone[Title/Abstract]) OR intra-operative parathormone[Title/Abstract]) OR parathyroid hormone monitoring[Title/Abstract]) OR parathyroid monitoring[Title/Abstract]) OR PTH monitoring[Title/Abstract]) OR Quick PTH[Title/Abstract]) OR QPTH[Title/Abstract]) OR Quick-Intraoperative[Title/Abstract]) OR intraoperative parathormone measurement[Title/Abstract]) OR intra-operative parathormone measurement[Title/Abstract]) OR Bio-Intact PTH[Title/Abstract]) OR intraoperative intact parathyroid hormone[Title/Abstract]) OR intraoperative iPTH[Title/Abstract]) OR Intact PTH[Title/Abstract]) OR Intact parathyroid hormone[Title/Abstract]) OR parathyroid hormone[MeSH Terms] | 31377 |
| #5 | #1 OR #2 | 311703 |
| #6 | #5 AND #3 = ((#1 OR #2) AND #3) | 17570 |
| #7 | #6 AND #4 = = ((#1 OR #2) AND #3) AND #4 | 1266 |

### Embase

|  |  |  |
| --- | --- | --- |
| #1 | ‘Renal hyperparathyroidism’:ab,ti OR ‘Renal HPT’:ab,ti OR ‘rHPT’:ab,ti OR ‘Secondary hyperparathyroidism’:ab,ti OR ‘Secondary HPT’:ab,ti OR ‘sHPT’:ab,ti OR ‘2HPT’:ab,ti OR ‘Tertiary hyperparathyroidism’:ab,ti OR ‘Tertiary HPT’:ab,ti OR ‘3HPT’:ab,ti OR 'secondary hyperparathyroidism'/exp | 12492 |
| #2 | ‘Chronic kidney disease’:ab,ti OR ‘Chronic kidney insufficiency’:ab,ti OR ‘CKD’:ab,ti OR ‘End stage kidney disease’:ab,ti OR ‘End-stage kidney disease’:ab,ti OR ‘End stage renal disease’:ab,ti OR ‘End-stage renal disease’:ab,ti OR ‘ESRD’:ab,ti OR ‘Renal insufficiency’:ab,ti OR ‘Dialysis’:ab,ti OR ‘Hemodialysis’:ab,ti OR ‘Haemodialysis’:ab,ti OR ‘Renal replacement therapy’:ab,ti OR ‘Kidney transplant’:ab,ti OR ‘Kidney transplantation’:ab,ti OR ‘Renal transplant’:ab,ti OR ‘Renal transplantation’:ab,ti OR 'chronic kidney failure'/exp | 445615 |
| #3 | ‘Parathyroidectomy’:ab,ti OR ‘Resection’:ab,ti OR ‘Surgery’:ab,ti OR ‘PTx’:ab,ti OR ‘parathyroidectomy’/exp | 1815279 |
| #4 | ‘ioPTH’:ab,ti OR ‘intraoperative PTH’:ab,ti OR ‘intra-operative PTH’:ab,ti OR ‘intraoperative parathyroid hormone’:ab,ti OR ‘intra-operative parathyroid hormone’:ab,ti OR ‘intraoperative parathormone’:ab,ti OR ‘intra-operative parathormone’:ab,ti OR ‘parathyroid hormone monitoring’:ab,ti OR ‘parathyroid monitoring’:ab,ti OR ‘PTH monitoring’:ab,ti OR ‘Quick PTH’:ab,ti OR ‘QPTH’:ab,ti OR ‘Quick-Intraoperative’:ab,ti OR ‘intraoperative parathormone measurement’:ab,ti OR ‘intra-operative parathormone measurement’:ab,ti OR ‘Bio-Intact PTH’:ab,ti OR ‘intraoperative intact parathyroid hormone’:ab,ti OR ‘intraoperative iPTH’:ab,ti OR ‘Intact PTH’:ab,ti OR ‘Intact parathyroid hormone’:ab,ti OR ‘parathyroid hormone’/exp | 56866 |
| #5 | #1 OR #2 | 450982 |
| #6 | #5 AND #3 = ((#1 OR #2) AND #3) | 31273 |
| #7 | #6 AND #4 = = ((#1 OR #2) AND #3) AND #4 | 2523 |
| #8 | #7 AND [embase]/lim | 2302 |

### Web of Science

|  |  |  |
| --- | --- | --- |
| #1 | TS=(Renal hyperparathyroidism OR Renal HPT OR rHPT OR Secondary hyperparathyroidism OR Secondary HPT OR sHPT OR 2HPT OR Tertiary hyperparathyroidism OR Tertiary HPT OR 3HPT) | 12494 |
| #2 | TS=( Chronic kidney disease OR Chronic kidney insufficiency OR CKD OR End stage kidney disease OR End-stage kidney disease OR End stage renal disease OR End-stage renal disease OR ESRD OR Renal insufficiency OR Dialysis OR Hemodialysis OR Haemodialysis OR Renal replacement therapy OR Kidney transplant OR Kidney transplantation OR Renal transplant OR Renal transplantation) | 382097 |
| #3 | TS=(Parathyroidectomy OR Resection OR Surgery OR PTx | 1262013 |
| #4 | TS=( ioPTH OR intraoperative PTH OR intra-operative PTH OR intraoperative parathyroid hormone OR intra-operative parathyroid hormone OR intraoperative parathormone OR intra-operative parathormone OR parathyroid hormone monitoring OR parathyroid monitoring OR PTH monitoring OR Quick PTH OR QPTH OR Quick-Intraoperative OR intraoperative parathormone measurement OR intra-operative parathormone measurement OR Bio-Intact PTH OR intraoperative intact parathyroid hormone OR intraoperative iPTH OR Intact PTH OR Intact parathyroid hormone) | 7493 |
| #5 | #1 OR #2 | 388311 |
| #6 | #5 AND #3 = ((#1 OR #2) AND #3) | 22988 |
| #7 | #6 AND #4 = = ((#1 OR #2) AND #3) AND #4 | 786 |

### Cochrane Reviews

|  |  |  |
| --- | --- | --- |
| #1 | (Renal hyperparathyroidism):ti,ab,kw OR (Renal HPT):ti,ab,kw OR (rHPT):ti,ab,kw OR (Secondary hyperparathyroidism):ti,ab,kw OR (Secondary HPT):ti,ab,kw OR (sHPT):ti,ab,kw OR (2HPT):ti,ab,kw OR (Tertiary hyperparathyroidism):ti,ab,kw OR (Tertiary HPT):ti,ab,kw OR (3HPT):ti,ab,kw | 2 |
| #2 | (Chronic kidney disease):ti,ab,kw OR (Chronic kidney insufficiency):ti,ab,kw OR (CKD):ti,ab,kw OR (End stage kidney disease):ti,ab,kw OR (End-stage kidney disease):ti,ab,kw OR (End stage renal disease):ti,ab,kw OR (End-stage renal disease):ti,ab,kw OR (ESRD):ti,ab,kw OR (Renal insufficiency):ti,ab,kw OR (Dialysis):ti,ab,kw OR (Hemodialysis):ti,ab,kw OR (Haemodialysis):ti,ab,kw OR (Renal replacement therapy):ti,ab,kw OR (Kidney transplant):ti,ab,kw OR (Kidney transplantation):ti,ab,kw OR (Renal transplant):ti,ab,kw OR (Renal transplantation):ti,ab,kw | 255 |
| #3 | (Parathyroidectomy):ti,ab,kw OR (Resection):ti,ab,kw OR (Surgery):ti,ab,kw OR (PTx):ti,ab,kw | 1730 |
| #4 | (ioPTH):ti,ab,kw OR (intraoperative PTH):ti,ab,kw OR (intra-operative PTH):ti,ab,kw OR (intraoperative parathyroid hormone):ti,ab,kw OR (intra-operative parathyroid hormone):ti,ab,kw OR (intraoperative parathormone):ti,ab,kw OR (intra-operative parathormone):ti,ab,kw OR (parathyroid hormone monitoring):ti,ab,kw OR (parathyroid monitoring):ti,ab,kw OR (PTH monitoring):ti,ab,kw OR (Quick PTH):ti,ab,kw OR (QPTH):ti,ab,kw OR (Quick-Intraoperative):ti,ab,kw OR (intraoperative parathormone measurement):ti,ab,kw OR (intra-operative parathormone measurement):ti,ab,kw OR (Bio-Intact PTH):ti,ab,kw OR (intraoperative intact parathyroid hormone):ti,ab,kw OR (intraoperative iPTH):ti,ab,kw OR (Intact PTH):ti,ab,kw OR (Intact parathyroid hormone):ti,ab,kw | 1 |
| #5 | #1 OR #2 | 255 |
| #6 | #5 AND #3 = ((#1 OR #2) AND #3) | 47 |
| #7 | #6 AND #4 = = ((#1 OR #2) AND #3) AND #4 | 0 |

## *Appendix S2: Modified QUIPS criteria*

**QUIPS tool component ‘Systematic Review intraoperative PTH measurements during parathyroidectomy for renal hyperparathyroidism’:**

1. **Study participation**
   1. Adequate participation
      1. Low risk: Consecutive cohort of patients with renalHPT and ioPTH measurement
      2. Moderate risk: Some excluded/not included (<10%), but described
      3. High risk: Selective sample (>10% exclusion)
   2. Source population
      1. Low risk: Clear description provided; including specific criteria for surgery for rHPT
      2. Moderate risk: Unclear description; definition of rHPT not defined
      3. High risk: No description provided
   3. Baseline study sample
      1. Low risk: All of the baseline characteristics* provided
      2. Moderate risk: Most baseline characteristics* provided; at least age, sex cause of CKD, whether the patient was operated with a functioning transplant or not
      3. High risk: No clear baseline characteristics*; any of the characteristics listed in “most baseline” missing from description

*Most important characteristics: age, sex, cause of CKD, GFR, preoperative PTH, RRT, transplant.

- 1. Period and place of recruitment
     1. Low risk: Described
     2. Moderate risk: Not described
  2. Inclusion/exclusion criteria
     1. Low risk: Described
     2. Moderate risk: Limited description
     3. High risk: Not described
  3. Overall risk of bias ‘Study participation’ domain:
     1. High: If selective population, i.e., exclusion/non-inclusion of many patients
     2. Moderate: If unclear description of inclusion, or only some patients excluded which are described, or baseline characteristics not described
     3. Low: Consecutive cohort of patient undergoing PTx for renal HPT with ioPTH and baseline characteristics described

1. **Study Attrition**
   1. Response rate
      1. Low risk: All patients followed
      2. Moderate risk: >80% patients followed
      3. High risk: <80% followed
   2. Attempts to gather info on FU
      1. Low risk: Described
      2. High risk: Not described
   3. Reasons loss to FU
      1. Low risk: Described
      2. High risk: Not described
   4. Description of lost to FU
      1. Low risk: Described, no major differences between completing and non-completing patients
      2. Moderate risk: Described, unclear differences between completing and non-completing patients
      3. High risk: Not described or clear differences between completing and non-completing patients
   5. Differences between dropouts
      1. Low risk: Described and small
      2. Moderate risk: Described and large
      3. High risk: Not described
   6. Overall risk of bias ‘Study Attrition’ domain:
      1. High: If selective sample analyzed (<80%) and patients analyzed differ from those who did not complete the study
      2. Moderate: 80-100% analyzed, but unclear regarding loss to FU OR <80% but clear description of those lost to FU and no selective loss to FU
      3. Low: 80-100% analyzed and information on loss to FU (if there is loss to FU), so no differences between patients who completed the study and those who did not
2. **Prognostic Factor Measurement**
   1. Clear definition of factor
      1. Low risk: Protocol describing all factors*
      2. Moderate risk: Protocol describing 3-6 factors
      3. High risk: Unclear protocol or <3 factors described

*Factors: type of assay (ELISA, IRMA, chemiluminescence Immunoassay), manufacturer of the assay, intact PTH (1-84) or fragments (7-84), serum or plasma measurement, CV for the assay, timing of measurement, type of sample (peripheral vein, jugular vein)

- 1. Valid and reliable method
     1. Low risk: Definition of quality control of the measurement*
     2. Moderate risk: Not described

*I.e., the lab takes part in a quality control program, or batches are calibrated

- 1. Continuous variables reported
     1. Low risk: Continuous PTH values reported
     2. Moderate risk: Cut-offs used, but no data driven cut-offs
     3. High risk: Not described or data driven cut-off used
  2. Same ioPTH method for all patients
     1. Low risk: Described and performed
     2. Moderate risk: Described, but unclear if performed in all patients
     3. High risk: Not described or not performed in all patients
  3. Adequate proportion of patients with complete data
     1. Low risk: Measured according to protocol in 100 %
     2. Moderate risk: Measured according to protocol in >80-100%
     3. High risk: Measured according to protocol in <80%
  4. Overall risk of bias ‘Prognostic Factor Measurement’ domain:
     1. High: Measurement method not described or different between patients
     2. Moderate: Measurement method not adequately described, but the same in all patients
     3. Low: Measurement method adequately described and performed the same in all patients

1. **Outcome Measurement**
   1. Clear definition of outcome
      1. Low risk: Method and timing described
      2. Moderate risk: Method described, but exact timing unclear
      3. High risk: Method not clearly described
   2. Method of outcome measurement reliable
      1. Low risk: If the same assay is used for postoperative PTH as intraoperative PTH, or the authors describe how these values can be compared/calibrated/corrected with each other
      2. Moderate risk: If different assays are used, and this is described, but no correlation/correction factor between the assays are given
      3. High risk: Not described or if it is unclear whether postoperative and intraoperative PTH were measured with the same or different assays
   3. Method and setting of outcome measurement is the same for all study participants
      1. Low risk: Outcome measure in the same way in all patients
      2. Moderate risk: Outcome measure in the same way in >80% patients
      3. High risk: Outcome measure in the same way in <80%, or measured selectively
   4. Overall risk of bias ‘Outcome Measurement’ domain:
      1. High: <80% with outcome data, or different measurement method or timing across patients
      2. Moderate: 80-100%, but same measurement method and timing
      3. Low: >95% and same measurement method and timing
2. **Confounders**
   1. All confounders measured*
      1. Low risk: Confounders measured
      2. Moderate risk: Most confounders measured.
      3. High risk: No confounders measured

* Age, sex, transplant/no transplant, cause of renal failure (diabetes, hypertension, etc), perhaps also renal function (GFR), perhaps also medication, previous surgery for rHPT, previous transplantation, dialysis vintage, duration of dialysis, type of surgery

- 1. Definitions of confounders
     1. Low risk: Definitions reported
     2. Moderate risk: Not all confounders are defined
     3. High risk: Not reported
  2. Measurement of confounders reliable
     1. Low risk: Reliable
     2. Moderate risk: Some confounders measured reliably
     3. High risk: Not reliable or unclear
  3. Same for all
     1. Low risk: Yes
     2. Moderate risk: Most
     3. High risk: No or unclear
  4. Overall risk of bias ‘Confounders’ domain:
     1. High: None or some confounders reported, but no definitions and measurement methods given
     2. Moderate: Most confounders reported and some definitions given
     3. Low: All confounders reported, definitions given and valid and reliable measurement methods used

1. **Statistical Analysis and Reporting**
   1. Sufficient presentation of data
      1. Low risk: Sufficient data provided; individual patient data analyzed
      2. Moderate risk: group level data with measure of dispersion
      3. High risk: Insufficient data provided
   2. No selective reporting
      1. Low risk: No
      2. Moderate risk: Probable
      3. High risk: Yes
   3. Overall risk of bias ‘Statistical Analysis and Reporting’ domain:
      1. High: Insufficient data presented to assess adequacy of analysis and selective reporting/spurious associations
      2. Moderate: Insufficient data, no selective reporting
      3. Low: Sufficient data presented, no selective reporting

## *Appendix S3: Excluded studies based on full text screening*

| **Exclusion reason full text, N=84** | **First author and year** |
| --- | --- |
| *No one month PTH levels, n=20* | |
| No one month PTH levels, n=17 | Roshan et al., 2006  Casarim et al., 2019  Cheung et al., 2011  Clary et al., 1997  Ermer et al., 2019  Freriks et al., 2010  Gasparri et al., 2009  Gilat et al., 2007  Kim et al., 2012  Konturek et al., 2016  Moor et al., 2011  Ohe et al., 2013  Pellitteri et al., 2003  Pitt et al., 2010  De Vos tot Nederveen et al., 2007  Stracke et al., 2009  Thanasoulis et al., 2007 |
| Unclear timing of outcome, n=3 | Milas et al., 2004  Somnay et al., 2014  Weber et al., 2005 |
| *Categorized PTH levels, n=9* | |
| Categorized PTH levels one month, n=9 | Bieglmayer et al., 2006  Hiramitsu et al., 2015  Kaczirek et al., 2005  Kaczirek et al., 2006  Ohe et al., 2003  Proctor et al., 2003  Yamashita et al., 2002  Yamashita et al., 2004  Yamashita et al., 2005 |
| *No postoperative PTH levels reported, n=7* | |
| No postoperative PTH levels, n=7 | Damiano et al., 2016  Giovale et al., 2007  Guarda, 2004  Ikeda et al., 2007  Ikeda et al., 2007  Giovale et al., 2006  Ryan et al., 1992 |
| *No intraoperative PTH levels reported, n=5* | |
| No intraoperative PTH levels reported, n=5 | Amza et al., 2017  Haustein et al., 2005  Mengozzi et al., 2000  Meyer et al., 2009  Neves et al., 2018 |
| *Publication type or availability, n=36* |  |
| Other language, n=11 | Cao et al., 2015  Duquenne et al., 1992  Gasparri et al., 2003  Koeberle-Wuehrer et al., 1999  Martin et al., 1997  Barczynski et al., 2003  Ohe et al., 2006  Pino Rivero et al., 2007  Pino Rivero et al., 2011  Pulgar et al., 2015  Romani et al., 2007 |
| Duplicate, n=8 | - |
| Conference abstract, n=7 | Kovacevic et al., 2012  Webb et al., 2013  Kwon et al., 2005  McFarlane et al., 2005  McFarlane et al., 2005  Schielen et al., 2001  Hiramitsu et al., 2014 |
| Other publication type, n=6 | Giovale et al., 2016  Lorenz and Dralle, 2005  Lorenz et al., 2015  Miccoli, 2012  Sokoll, 2004  Hiramitsu et al., 2015 |
| No full text available, n=4 | Arciero et al., 2004  Portakal et al., 2017  Proye et al., 1991  Niramitmahapanya et al., 2018 |
| *Other, n=7* | |
| Less than 5 patients with rHPT, n=2 | Halevy et al., 2003  Stratmann et al., 2002 |
| Combined with pHPT, n=2 | Patel et al. 1998  Starr et al. 2000 |
| Other, n=3  No parathyroid surgery, n=1  Autotransplant recurrence, n=1  Bilateral internal jugular venous sampling for parathyroid localization, n=1 | Sippel et al., 2004  Agha et al. 2012  Ito et al., 2007 |
