# Supplementary material for: Use of intraoperative parathyroid hormone measurements during parathyroidectomy to predict postoperative parathyroid hormone levels in patients with renal hyperparathyroidism: meta-analysis
Source: BJS Open. 2022 Feb 15;6(1):zrab151. doi: 10.1093/bjsopen/zrab151 (PMC8855528; doi:10.1093/bjsopen/zrab151)
Supplement: zrab151_Supplementary_Data [file zrab151_supplementary_data.zip › Supplementary_Table_1.docx]

## *Table S1. Patient characteristics of patients with a renal transplant*

| **Supplementary Table 1. Patient characteristics of patients with a renal transplant** | | | | |
| --- | --- | --- | --- | --- |
| **Study** | **No. pts** | **Time renal transplant PTX in months** | **eGFR (ml/min)** | **Creatinine (μmol/l)** |
| El-Husseini^44^ | 18 | 33.7 ± 52.3^*^ | 51.7 ± 16.1^*^ | 123.8 ± 35.4^*^ |
| Müller-Stich^48^ | 7 | n.r. | 58.53 ± 15.94^*^ | n.r. |
| Seehofer^50^ | 24 | n.r. | n.r. | 185.6 ± 172.4^§^ |
| Triponez^54^ | 35 | 63.6 ± 61.2 | ﻿53 ± 21;  20 pts <60 ml/min and 15 pts >60 ml/min | n.r. |
| All continuous variables presented as mean ± standard deviation.  ^§^Mean ± standard error of the mean was converted to mean ± standard deviation based on the formula reported in the Cochrane handbook.^33^  ^*^Data were transformed from non-normally distributed data (i.e., median + interquartile range/range) into mean ± standard deviation based on the formulas proposed by Wan et al.^34^.  Abbreviations: *eGFR* estimated glomerular filtration rate, *No.* number of, *n.r.* not reported, *P* phosphate, *PTH* parathyroid hormone, *pts* patients, *PTX* parathyroidectomy | | | | |
