# Supplementary material for: Use of intraoperative parathyroid hormone measurements during parathyroidectomy to predict postoperative parathyroid hormone levels in patients with renal hyperparathyroidism: meta-analysis
Source: BJS Open. 2022 Feb 15;6(1):zrab151. doi: 10.1093/bjsopen/zrab151 (PMC8855528; doi:10.1093/bjsopen/zrab151)
Supplement: zrab151_Supplementary_Data [file zrab151_supplementary_data.zip › Supplementary_Table_2.docx]

## *Table S2. PTH assays*

| **Supplementary Table 2. PTH Assays used** | | | | | | | | | | |
| --- | --- | --- | --- | --- | --- | --- | --- | --- | --- | --- |
|  | **Intraoperative PTH** | | | | | **Postoperative PTH** | | | | |
| **Study** | **PTH** | **Assay** | **Manufacturer** | **CoV** | **Ref. range** | **PTH** | **Assay** | **Manufacturer** | **CoV** | **Ref. range** |
| Barczynski^21^ | iPTH | ICMA | STAT intraoperative intact-PTH ICMA (Future Diagnostics) |  |  |  | IRMA | Traditional 24-h IRMA in Central Laboratory |  | 1.06 – 6.89 pmol/l^§^ |
| Chou^40^ | iPTH | IRMA | Rapid PTH |  |  | iPTH | IRMA | 2-site IRMA (Nichols Institute Diagnostics) |  |  |
|  | iPTH | IRMA | 2-site IRMA (Nichols Institute Diagnostics) | Intra 1.7–3.7%;  inter 2.6–5.9% |  |  |  |  |  |  |
| Conzo^41^ | iPTH | ICMA | ICMA (Liaison N-Tact PTH Assay [DiaSorin Inc]) |  | 1.06 - 6.89  pmol/l | iPTH | ICMA | ICMA (Liaison N-Tact PTH Assay [DiaSorin Inc.) | Intra 1.7–3.7%;  Inter 2.6–5.9% | 1.06 - 6.89 pmol/l |
| Echenique^42^ | iPTH | ICMA | Immuno-analyzer Immulite-turbo (DPC-Dipesa |  | 1.59 – 6.89  pmol/l^§^ |  |  |  |  |  |
| El-Husseini^44^ | whole PTH |  | Quick-Intraoperative Bio-Intact PTH assay (Nichols Institute Diagnostics) |  |  | iPTH | ICMA | Standard ICMA (Elecsys System, Roche) | Intra 1<6%;  Inter <9 | 1.59 - 6.89 pmol/l^§^ |
| Kara^45^ | iPTH | ICMA | Bio-iPTH (Nichols Institute Diagnostics) |  | 1.27 – 9.33  pmol/l^§^ | iPTH | ICMA | Bio-iPTH (Nichols Institute Diagnostics) |  | 1.27 – 9.33 pmol/l^§^ |
| Lorenz^46^ |  | ICMA | qPTH Commercially available 2-site-antibody assay |  | 1.27 – 7.63  pmol/l^§^ |  |  |  |  |  |
| Matsuoka^47^ | iPTH (1-84) | ICMA | QuiCk-Intra Operative Bio-Intact PTH (1-84) assay (QPTH) (Nichols Institute Diagnosis) |  |  | iPTH |  |  |  |  |
| Müller-Stich^48^ | iPTH | ICMA | IMMULITE Turbo Intact PTH Assay (Diagnostic Products Corporation) |  | 1.59 – 8.48  pmol/l^§^ | iPTH | ICMA | IMMULITE Turbo Intact PTH Assay or ICMA Elecsys 2010 (Roche) |  | 1.59 – 6.89 pmol/l^§^ |
| Seehofer^50^ | iPTH |  | Roche Elecsys 2010 system (Roche) |  | 1.59 – 6.89  pmol/l^§^ | iPTH |  | Elecsys 2010 system (Roche) |  | 1.59 – 6.89 pmol/l^§^ |
| Triponez^54^ | 1-84 PTH,  7-84 PTH |  | Nichols Institute Diagnostics |  | 1.06 – 6.36  pmol/l^§^ | iPTH | IRMA |  |  | 1.06 – 6.36 pmol/l^§^ |
| Vulpio^51^ | iPTH | ICMA | Roche Intact PTH assay running on a Roche Modular E 170 analyzer | Intra 2.9%; Inter 5.8%* | 1.59 – 6.89  pmol/l^§^ | iPTH | ICMA | 2nd generation iPTH ICMA (Roche Intact PTH) and Roche Modular E 170 analyzer | Intra 2.9%;  Inter 5.8%* | 1.59 – 6.89 pmol/l^§^ |
| Walgenbach^52^ | iPTH |  | Rapid Assay (Elecsys, Roche) |  |  | iPTH | IRMA | PTH-IRMA Medgenix Group |  |  |
| Zhang^53^ | iPTH |  | UniCel DxI800 Access Immunoassays System (Beckman Coulter) |  | 1.27 – 9.33  pmol/l^§^ | iPTH |  | UniCel DxI800 Access Immunoassays System (Beckman Coulter) |  | 1.27 – 9.33 pmol/l^§^ |
| ^§^Data were converted from pg/ml into pmol/l (pg/ml times 0.106).^15^  *At 35.0 and 180.0 ng/l, respectively.  Abbreviations: *CoV* coefficient of variation, *iPTH* intact PTH, *ICMA ﻿*immunochemiluminometric Assay*, IRMA* ﻿immunoradiometric Assay, *PTH* parathyroid hormone | | | | | | | | | | |
