# Supplementary material for: Use of intraoperative parathyroid hormone measurements during parathyroidectomy to predict postoperative parathyroid hormone levels in patients with renal hyperparathyroidism: meta-analysis
Source: BJS Open. 2022 Feb 15;6(1):zrab151. doi: 10.1093/bjsopen/zrab151 (PMC8855528; doi:10.1093/bjsopen/zrab151)
Supplement: zrab151_Supplementary_Data [file zrab151_supplementary_data.zip › Supplementary_Table_3.docx]

## *Table S3. Mean percentage decrease of preoperative PTH.*

| **Supplementary Table 3. Mean percentage of induction or preoperative PTH.** | | | | | | | |
| --- | --- | --- | --- | --- | --- | --- | --- |
| **Study** | **T0 (%)^\|^** | **T5 (%)** | **T10 (%)** | **T15 (%)** | **T20 (%)** | **T30 (%)** | **Postop (%)** |
| *Patients on dialysis* | | | | | | | |
| Barczynski^21^ |  |  | 21.2^¶^ |  |  |  | 1.9^¶^ |
| Chou^40^ | 48.3^§^ |  | 17.8^§^ |  |  | 9.7^§^ | 2.2^§^ |
| Conzo^41^ |  |  | 41.6^§¶^ |  | 11.2^§¶^ |  | 3.7^§¶^ |
| Echenique^42^ | 107.0^*^^ | 65.7^*^^ | 45.7 | 10.1^*^^ | 6.0^*^^ | 4.8^*^^ | 0.7 |
| El-Husseini^44^ |  |  | 10.2^*^ |  | 7.1^*^ |  | 1.5^*^^ |
| Kara^45^ |  |  |  | 10.0^§¶^ |  |  | 5.4^¶^^ |
| Lorenz^46^ |  |  | 8.4 |  | 8.0 | 0.8 | 9.0 |
| Matsuoka^47^ | 14.1 | 7.9 | 5.0 | 3.6 |  | 2.2 | 1.8 |
| Müller-Stich^48^ |  |  | 12.8^*¶^ |  |  |  | 3.1^*¶^ |
| Seehofer^50^ | 102.2^¶§^ |  |  | 19.9^¶§^ |  |  | 5.1^¶§^ |
| Vulpio^51^ | 101.1 |  | 26.5^*¶^^ |  | 14.4^*¶^^ | 11.5^*¶^^ | 2.1^*¶^^ |
| Walgenbach^52^ |  | 21.7 |  |  |  |  | 5.0 |
| Zhang^53^ |  |  | 14.2^*¶^^ |  | 11.1^*¶^^ |  | 3.0^*¶^ |
| **Weighted mean** | **84.6** | **22.6** | **17.2** | **14.1** | **10.6** | **6.0** | **3.2** |
| *Patients with a functioning kidney transplant* | | | | | | | |
| El-Husseini^44^ |  |  | 16.2^*^ |  | 11.9^*^ |  | 26.1^*^^ |
| Müller-Stich^48^ |  |  | 5.0^*¶^ |  |  |  | 5.2^*¶^ |
| Seehofer^50^ | 105.0^¶§^ |  |  | 16.8^¶§^ |  |  | 6.9^*¶^ |
| Triponez^54^ |  | 22.1^*^^ | 18.5^*^^ |  | 14.9^*^^ | 13.4^*^^ | 4.6 |
| **Weighted mean** | **105.0** | **22.1** | **16.3** | **16.8** | **13.9** | **13.4** | **9.9** |
| ^\|^T0 surgery levels were reported, before resection of last parathyroid gland (Conzo *et a*l.^41^), at cut time (Echenique *et al*.^42^), immediately after removal of the last gland (Matsuoka *et al*.^47^), after skin incision and exposure of the thyroid, but before preparation of the parathyroid glands (Seehofer *et al*.^50^).  ^§^Mean ± standard error of the mean was converted to mean ± standard deviation based on the formula reported in the Cochrane handbook.^33^  ^¶^Studies reported values within two subgroups which were combined based on the formulae reported in the Cochrane handbook.^33^ If studies reported non-normally distributed values, these were transformed to mean ± standard deviation first.  ^*^Data were transformed from non-normally distributed data (i.e., median + interquartile range/range) into mean ± standard deviation based on the formulas proposed by Wan *et al*.^34^.  ^^^Data were extracted from figures.  Abbreviations: *PTH* parathyroid hormone, *T* time point | | | | | | | |
